# Demographic Representation of Generative Artificial Intelligence Images of Physicians

**DOI:** 10.1001/jamanetworkopen.2024.25993

**Published:** 2024-08-06

**Authors:** Sang Won Lee, Mary Morcos, Dong Won Lee, Jason Young

**Affiliations:** 1 Harvard Medical School, Boston, Massachusetts; 2 Nova Southeastern University Kiran C. Patel College of Osteopathic Medicine, Davie, Florida; 3 Harvard Combined Orthopedic Residency Program, Boston, Massachusetts

## Abstract

This cross-sectional study compares the race and ethnicity and gender of images of physicians generated by artificial intelligence (AI) with US physician demographic characteristics.

## Introduction

Generative artificial intelligence (AI) is a powerful tool that has revolutionized economic outlooks across multiple sectors, including health care. As text-to-image generative AI has become popular, its applications in health care have the potential to amplify racial, ethnic, and gender biases.^1,2^ This concern is particularly alarming and may undermine ongoing diversity, equity, and inclusion (DEI) initiatives within clinical and medical education landscapes.^3^ Consequently, we performed a demographic evaluation of AI-generated images of physicians and compared them with those of physicians in the US. We aimed to assess the implications of text-to-image generative AI on demographic representation for physicians as a first step toward understanding the role of generative AI in health care.

## Methods

We used the Harvard Longwood Campus Institutional Review Board Decision Tool to determine exemption for nonhuman research. Images of physicians were generated across 5 popular AI text-to-image platforms: DALL-E 2 (platform 1; Open AI; images created on February 1, 2024), Imagine AI Art Generator (platform 2; Vyro AI; February 2, 2024), Jasper Art: AI Art Generator (platform 3; Jasper AI; February 2, 2024), Midjourney Beta (platform 4; Midjourney; March 19, 2024), and Text-to-Image (platform 5; Runway AI; February 2, 2024). For each platform, 50 images were created for each of the following search terms: “face of a doctor in the United States,” “face of a physician in the United States,” “photo of a doctor in the United States,” and “photo of a physician in the United States” for a total of 1000 images across 5 platforms. Images with partial or multiple identifiable faces were excluded, and new images were generated. Racial and ethnic categories (White, Black, Asian [including East Asian and South Asian], Latino, and unable to determine or other) were developed based on validated classification systems, including the Chicago Face Dataset, IBM Diversity in Faces, and UTKFace.^4^ Two independent raters (M.M. and D.W.L.) evaluated each image for race and ethnicity and gender, with resolution of differential classification by group consensus. We compared the distribution of race and ethnicity and gender within each platform and combined across all platforms with US physician demographics based on the 2023 Association of American Medical Colleges (AAMC) survey.^5^ Comparisons across groups were conducted using χ^2^ tests with a Bonferroni correction for multiple hypothesis testing. Interrater reliability was assessed using Cohen κ. The α level was set at .05. All statistical tests were performed in R, version 4.2.3. This cross-sectional study followed the STROBE reporting guideline.

## Results

Overall, AI-generated images of physicians were more frequently White (82% vs 63%; *P* < .001) and more frequently men (93% vs 62%; *P* < .001) compared with the US physician population. All 5 platforms individually had significant differences in the distribution of race and ethnicity and gender compared with the AAMC census (*P* < .001). However, there was variability in the distribution of race and ethnicity and gender across platforms, with 3 platforms producing no images of Latino physicians, 2 platforms producing no images of Asian physicians, and 1 platform producing no images of female physicians (Table). Interrater reliability between the graders for race and ethnicity and gender classifications was κ = 0.81 and κ = 0.88, respectively.

**Table.** Race and Ethnicity and Gender of Artificial Intelligence–Generated Images of Physicians

Physician	Artificial intelligence–generated platforms						AAMC survey, total (n = 886 030)^b^
	Platform 1 (n = 200)^a^	Platform 2 (n = 200)^a^	Platform 3 (n = 200)^a^	Platform 4 (n = 200)^a^	Platform 5 (n = 200)^a^	Total (n = 1000)^a^	
Race and ethnicity, No. (%)							
Asian	4 (2)	0	29 (15)	11 (6)	0	44 (4)	186 175 (21)
Black	9 (5)	9 (5)	57 (29)	24 (12)	16 (8)	115 (12)	50 965 (6)
Latino	4 (2)	0	0	7 (4)	0	11 (1)	62 667 (7)
White	180 (90)	190 (95)	108 (54)	158 (79)	184 (92)	820 (82)	558 938 (63)
Other^c^	3 (2)	1 (1)	6 (3)	0	0	10 (1)	27 285 (3)
Gender, No. (%)^d^							
Women	42 (21)	0	5 (3)	16 (8)	11 (6)	74 (7)	371 851 (38)
Men	158 (79)	200 (100)	195 (98)	184 (92)	189 (95)	926 (93)	613 974 (62)

Abbreviation: AAMC, Association of American Medical Colleges.

^a^ Adjusted *P* < .001 following Bonferroni correction.

^b^ The unknown racial and ethnic category was removed in the AAMC survey data for a more accurate representation of distribution of race and ethnicity in the US physician workforce.

^c^ Defined as racial and ethnic categories not otherwise captured by Asian, Black, Latino, or White categories.

^d^ The “other” category was removed to fit the data categories available for physician demographics (women, men). Note that the total number for the gender section is 985 825.

## Discussion

Our study identifies demographic biases in AI-generated images of physicians with disproportionate representation of White and male physicians and concerning underrepresentation of other races and ethnicities (Asian and Latino) and female physicians in some platforms. This bias has the potential to reinforce stereotypes and undermine DEI initiatives within health care.^1^

Although strides toward a more representative health care workforce are being made as trainees from increasingly diverse backgrounds enter the workforce, this representation remains lacking within generative AI, highlighting a critical area for improvement.^6^ These biases necessitate closer examination of the training data and algorithms used by AI platforms. Ensuring a balanced depiction of the increasingly diverse workforce is essential to prevent AI from perpetuating existing inequities.

Future work should focus on enhancing training dataset diversity, creating algorithms capable of generating more representative images, while educating AI developers and users about the importance of diversity and inclusivity in AI output. By tackling these biases, AI can become a powerful tool for advancing DEI initiatives, rather than hindering it. This nuanced understanding of AI’s capabilities and limitations is critical because its use continues to grow in health care and beyond.

Limitations of the study include subjective assessment of race and ethnicity and gender of the included images. Two independent raters, a validated image classification,^4^ and interrater reliability testing were used to mitigate misclassification. Additionally, confinement of search terms to physician representation limits generalizability to other clinician classes, such as nurses and residents.

## Notes

Supplement.
Data Sharing Statement
