# Supplementary material for: Demographic Representation of Generative Artificial Intelligence Images of Physicians
Source: JAMA Netw Open. 2024 Aug 6;7(8):e2425993. doi: 10.1001/jamanetworkopen.2024.25993 (PMC11304108; doi:10.1001/jamanetworkopen.2024.25993)
Supplement: Supplement. — Data Sharing Statement [file jamanetwopen-e2425993-s001.pdf]

Data Sharing Statement

Lee. Demographic Representation of Generative Artificial Intelligence Images of Physicians. *JAMA Netw Open*. Published August 09, 2024. doi:10.1001/jamanetworkopen.2024.25993

Data
Data available: Yes
Data types: Data (not involving human participants)
How to access data: Requests can be sent to johnlee10@gmail.com
When available: With publication

Supporting Documents
Document types: None

Additional Information
Who can access the data: Anyone requesting the data
Types of analyses: For any purpose
Mechanisms of data availability: Without investigator support
